# Cow Milk Lactoferrin Possesses Several Catalytic Activities

**DOI:** 10.3390/biom9060208

**Published:** 2019-05-29

**Authors:** Svetlana E. Soboleva, Sergey E. Sedykh, Ludmila I. Alinovskaya, Valentina N. Buneva, Georgy A. Nevinsky

**Affiliations:** Institute of Chemical Biology and Fundamental Medicine of SB RAS, 8 Lavrentiev Ave., Novosibirsk 630090, Russia; sb543@ngs.ru (S.E.S.); sirozha@gmail.com (S.E.S.); sedyh@niboch.nsc.ru (L.I.A.); buneva@niboch.nsc.ru (V.N.B.)

**Keywords:** cow milk lactoferrin, multiple enzymatic activities, peroxidase, protease, amylase, phosphatase, ATPase

## Abstract

Lactoferrin (LF) is a Fe^3+^-binding glycoprotein, that was first recognized in milk and then in other epithelial secretions and barrier body fluids to which many different functions have been attributed to LF including protection from iron-induced lipid peroxidation, immunomodulation, cell growth regulation, DNA and RNA binding, as well as transcriptional activation, etc. The polyfunctional physiological role of LF is still unclear, but it has been suggested to be responsible for primary defense against microbial and viral infections. It was shown previously that human milk LF possesses several enzymatic activities: DNase, RNase, ATPase, phosphatase, and amylase. Analysis of human, cow, horse, buffalo and camel LF showed a highly conserved three-dimensional (3D) structure including only detail differences in the species. Recently, it was shown that similar to human cow LF possesses DNase and RNase activities. Using different methods here we have shown for the first time that LFs from the milk of seven cows of different breeds possess high peroxidase, protease, amylase, protease, and phosphatase activities. Protease activity of cow LFs was activated by Mg^2+^ and Ca^2+^ ions. In contrast to human LFs, ATPase activity was revealed only in three of seven cow LF preparations. The discovery that LF possesses these activities may contribute to understanding the multiple physiological functions of this extremely polyfunctional protein including its protective role against microbial and viral infections.

## 1. Introduction

Lactoferrin (LF) of mammal milk blood and other epithelial secretions and fluids is the protein consisting of a single polypeptide chain of ~80 kDa, containing two lobes [1], and each lobe contains one glycan chain and binds one Fe^3+^ ion [2]. Analysis of cow, human, horse, buffalo and camel LF showed a highly conserved three-dimensional (3D) structure including only detail differences in the species [3].

Many various biological functions were revealed for LF of human and cow milk including immunomodulation, cell growth regulation and protection from iron-induced lipid peroxidation [4,5]. Lactoferrin activities associate with primary antiviral and antibacterial effects [6,7], regulation of immune function [6,7,8], are important in antitumor defense [9], and may prove downgrade the risks of chronic human diseases [10]. Lactoferrin is known as the acute phase protein; the protein highest concentration is usually revealed in the inflammatory nidus. Lactoferrin is found in the neonate’s blood several hours after food intake, and it can penetrate facilely through different membranes of cells and nuclear [11]. It is believed that LF antimicrobial properties exist due to its iron-binding capacity [12,13,14]. Many different microorganisms express LF receptors on their surface, and this protein demonstrates various antiviral and antimicrobial iron-independent properties [15,16]. Polyfunctional LF due to antimicrobial, antiviral and many other activities can increase the passive immunity of neonates. Oral administration of bovine LF leads to host protection of patients from infections [13,14].

Human LF can bind and cleavage DNA [17,18,19,20,21] and RNA [19,22,23,24], and it activates specific DNA sequences as a specific transcriptional factor [22]. Human LF binds different nucleotides demonstrating ATPase [19,25,26] and phosphatase activities; it binds oligo- and polysaccharides demonstrating amylase activity [19]. Human LF is cytotoxic and induces apoptosis of tumor cells [19]. The human LF binding with ATP leads to changes of its structure and as a consequence to alteration of interaction with DNA, polysaccharides, and proteins [26]. Human LF possesses two different sites of DNA binding demonstrating anti-cooperative behavior during recognition of specific and non-specific DNAs [18,21]. Thus, human LF is a very polyfunctional protein and enzyme, which several enzymatic activities may expand its different biological functions including protection against microbial and viral infection [12,18,21].

It should be noted that cow LF is noticeably less investigated than human lactoferrin. Lactoferrin having many different biological functions has attracted growing scientific and commercial interests especially for clinical trials (for review see [7,10,27,28,29,30,31,32,33]). However, women’s milk is significantly less available than cow’s milk. Therefore, for medical purposes, cow lactoferrin is more suitable. We recently showed that, like female, bovine lactoferrin also possesses DNase and RNase activities [34]. On average, the DNase and RNase activities of bovine LF are about 1.5–2.0-fold lower than that of human milk.

The complete mRNA sequence of cow lactoferrin has high homology with that for human LF. At the same time, there are regional differences in the amino acid sequences [27]. Thus, different biological properties, including several enzymatic activities, human and cow LFs may be different in varying degrees. To date, in the literature there is no direct evidence of the role of the enzymatic activities of human and cow lactoferrins in the manifestation of their many known biological functions. There is only the data that catalytic activities of human LF may contribute to its protective functions due to LF-dependent hydrolysis of alien components of viruses and bacteria [35,36,37]. Here, we analyzed for the first time several enzymatic activities of cow milk LF. Using different methods, it was shown that milk LF of cows of different breeds possesses peroxidase, protease, amylase, phosphatase, and ATPase activities.

## 2. Materials and Methods

### 2.1. Materials and Chemicals

Reagents used in this work were mainly obtained from Sigma and Merck. DEAE-cellulose (DE-52; Whatman, Little Chalfont, Buckinghamshire, UK), SP-Sepharose and maltoheptaose (Sigma-Aldrich, St. Louis, MO, USA), and Superdex 200 HR 10/30 (GE Healthcare Life Sciences, Chicago, IL, USA). 

### 2.2. Purification and Analysis of Lactoferrin

Fresh milk from seven cows (200 mL from each cow) of several different Siberian breeds (Novosibirsk region, Russia) was centrifuged twice for 15 min at 6000× *g* for removing of fat and cells. The casein was removed using 3 M NaAc buffer (pH 4.0), which was added to the final 50 mM concentration with constant stirring of the solution. Then, (NH_4_)_2_SO_4_ was added to the final 33% saturation of the solution, which was kept at +4 °C during the night; the precipitate obtained was removed by centrifugation at 6000× *g* for 15 min. The solution obtained was again saturated with (NH_4_)_2_SO_4_ to 60–70% and was kept at +4 °C for 4 h, and the precipitate was removed as described above. The supernatant was diluted two times with 50 mM sodium-phosphate buffer (pH 7.7), and applied to 5 mL column with SP-Sepharose, equilibrated with the same sodium-phosphate buffer. The column has been washed with the same buffer (25 mL). Specifically bound LF was eluted with 15 mL of 50 mM sodium-phosphate buffer (pH 7.7) containing 0.3 M NaCl. The LF fractions were combined and dialyzed against Tris-HCl (20 mM; pH 7.5). 

Lactoferrin preparations were subjected to additional purification by fast protein liquid chromatography (FPLC) gel filtration on Superdex 200 according to [34]. The final preparations after gel filtration were additionally purified on Sepharose with immobilized rabbit antibodies against cow lactoferrin similar to [35] Anti-LF-Sepharose (20 × 1 cm), was equilibrated with 50 mM Tris-HCl, pH 7.5. After applying the preparation to the column, the sorbent was washed with 50 mM Tris-HCl, pH 7.5, containing 1.0 NaCl until the optical absorption disappeared. Elution of LF was performed with 50 mM Gly-HCl buffer, pH 2.6. The isolated protein fractions were neutralized with 1 M Tris-HCl, pH 8.0. The homogeneity of preparations was analyzed by sodium dodecyl sulfate-polyacrylamide gel electrophoresis (SDS-PAGE) according to Lemmley [37] and proteins were stained with silver. Estimation of LF concentration was performed using the standard Lowry method [38]. 

### 2.3. Analysis of Oxidoreductase Activities of Lactoferrin

Analysis of oxidoreductase activity was performed as in [39]. The reaction mixture (100–200 μL) for peroxidase activity analysis consisted of 25 mM K-phosphate (pH 6.8), 10 mM H_2_O_2_, 0.2 mg/mL 3,3’-diaminobenzidine (DAB) and 1 µM LF. The reaction mixture was incubated in immunological cells plates in the dark at 22 °C for 0.5–20 min. The optical density of the solutions (∆A_450_) was determined using a Multiscan FC Microplate Photometer (Thermo Fischer Scientific, Waltham, MA, USA). The reaction mixtures containing no LF were used as controls. The initial reaction rates were found from the slopes of the linear sections of the kinetic curves. The activity was first expressed in units of ∆A_450_/min/mg LF, and then recalculated in µmol DAB/h/mg LF using the absorbance of the oxidation product of DAB equal to 2807 units A_450_/1 M/1 cm [39]. 

### 2.4. Amylase Activity of Cow Lactoferrin

Amylase activity of LF was carried out as in [19]. Reaction mixtures (20–30 µL) contained 30 mM Tris-HCl (pH 7.5), 3 mM maltoheptaose (MHO), and 1 μM LF; they were incubated for 12–24 h at 37 °C. The products of substrate hydrolysis were identified by thin layer chromatography (TLC) on Kieselgel 60 plates (Merck, Darmstadt, Germany; ethanol-butanol-H_2_O; 2:2:1). The plates were dried, treated with 5% H_2_SO_4_ in 1-isopropanol and then dried at 110 °C to visualize the carbohydrates [19]. The activity was defined as µmol MHO/1 h/1 mg LF.

### 2.5. ATPase Activity Assay

ATPase activity of LF was analyzed as in [19]. Reaction mixtures (10–20 µL) containing 50 mM Tris-HCl, pH 6.8, 1.0 mM MgCl_2_, 0.3 mM ATP, 5 µM LF were incubated for 48 h at 37 °C. The ATP hydrolysis products were analyzed by thin-layer chromatography in 0.25 M KH_2_PO_4_ buffer, pH 7.0, on fluorescent PEI-cellulose plates (Merck). The plates were dried: The positions of adenosine-5’-dophosphate (ADP) were identified using its absorption (dark spots on a uniformly fluorescent plate background). The intensity of the spots of the initial ATP and product of its hydrolysis ADP was evaluated using scanning and then quantified by GelPro v3.1 software (Media Cybernetics, Bethesda, MD, USA). At first, ATPase activity was evaluated from the transition of ATP to ADP (%), and then recalculated to µmol ATP/1 h/1 mg LF taking into account the initial concentration of ATP (0.3 mM) and LF.

### 2.6. Phosphatase Activity Assay

For analysis of phosphatase activity the reaction mixture (80 μL) contained 20 mM Tris HCl, pH 9.0, 10 mM MgCl_2_, 5 mM *para*-nitrophenyl phosphate (*p*NPP), and 12.5 µM one of LF preparation. The accumulation of the colored product was measured at a wavelength of 400 nm for 1–6 min according to [40]. The specific activities of LFs were calculated from an increase in optical density at 400 nm (∆A_400_), and specific activity was expressed as M *p*NPP/h/mg of LF using the extinction coefficient equal to 18,300 M^−1^ sm^−1^ [40].

### 2.7. Protease Activity Assay

The reaction mixture (1.4 mL) contained azocasein (5 mg/mL), LF (0.27 µM) as well as in several experiments CaCl_2_ and MgCl_2_ were added in different concentrations (0–20 mM). The reaction was started by the addition of LF and the mixtures were incubated at 37 °C for 10–30 min. The reaction was stopped by adding of 75 μL of 20% trichloroacetic acid solution, centrifuged for 1 min at 12,000× *g*. 1 M NaOH (75 μL) was added to the supernatant and it was incubated at room temperature for 30 min. All samples were then centrifuged at 12,000× *g* for 3 min, the supernatant was taken and the absorbance (A_436_) was measured.

### 2.8. In-Gel Assays of Enzymatic Activities

Analysis of catalytic activities activity of cow LFs using SDS-PAGE was performed as in [19]. Several activities were determined using 4–15% SDS-PAGE gels. Lactoferrin preparations were incubated in 50 mM Tris-HCl, pH 6.8, buffer containing 1% SDS, 10% glycerol, 0.025% bromophenol blue at 37 °C for 15 min, and then applied to the gel. After SDS-PAGE, part of the gel was separated and stained with Coomassie R-250. The gel of the test run was washed from SDS for 1 h with a solution of 4 M urea and water (10 changes of water for 5–7 min each), after which the longitudinal strips of the gel were cut into pieces 2–3 mm in length and placed in separate tubes and crushed thoroughly. For renaturation of LF and restoration of their catalytic activities, pieces of the gel were incubated in 50 mM Tris-HCl, pH 7.5 containing 50 mM NaCl (150 μL) for 3–6 days at 4 °C. The gel was removed by centrifugation, and the supernatant (20–30 μL) was used to determine different catalytic activities as described above.

### 2.9. Statistical Analysis

The results are reported as a mean ± standard deviation of at least three independent experiments. Errors in the values were within 7–12%. 

## 3. Results

### 3.1. Purification and Characterization of Lactoferrin Preparations

**Figure 1:**
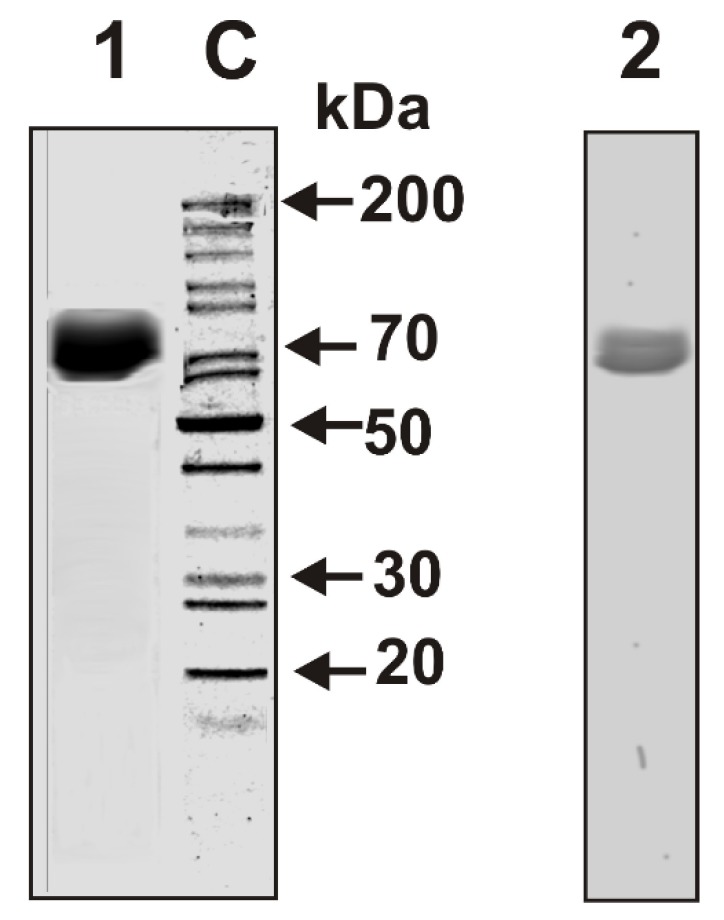
Sodium dodecyl sulfate-polyacrylamide gel electrophoresis (SDS-PAGE) analysis of LF_mix_ (8 µg) homogeneity from cow milk in 4–18% gradient gel followed by silver staining (**1**). Western blot of LF_mix_ using polyclonal mouse antibodies (Abs) against cow lactoferrin (LF) (**2**). Lane **C** shows the position of protein markers.

We have isolated individual LF preparations from seven cow milk of different breeds of the Novosibirsk region (Novosibirsk, Russia). Electrophoretically homogeneous LF preparations were isolated by sequential chromatography of milk proteins on DEAE-cellulose, SP-Sepharose, and then by FPLC gel filtration on Superdex 200. In addition, after gel filtration LF was then additionally purified by affinity chromatography on Sepharose with immobilized rabbit antibodies against cow lactoferrin. To analyze an “average” situation concerning electrophoretic and immunological homogeneity of lactoferrins, the mixture of equal amounts of seven LFs (LF_mix_) was prepared. The homogeneity of LF_mix_ was shown by SDS-PAGE with silver staining (Figure 1, lane 1) and by Western blot (Figure 1, lane 2). 

### 3.2. Sodium Dodecyl Sulfate-Polyacrylamide Gel Electrophoresis Analysis of Lactoferrin Enzymatic Activities

**Figure 2:**
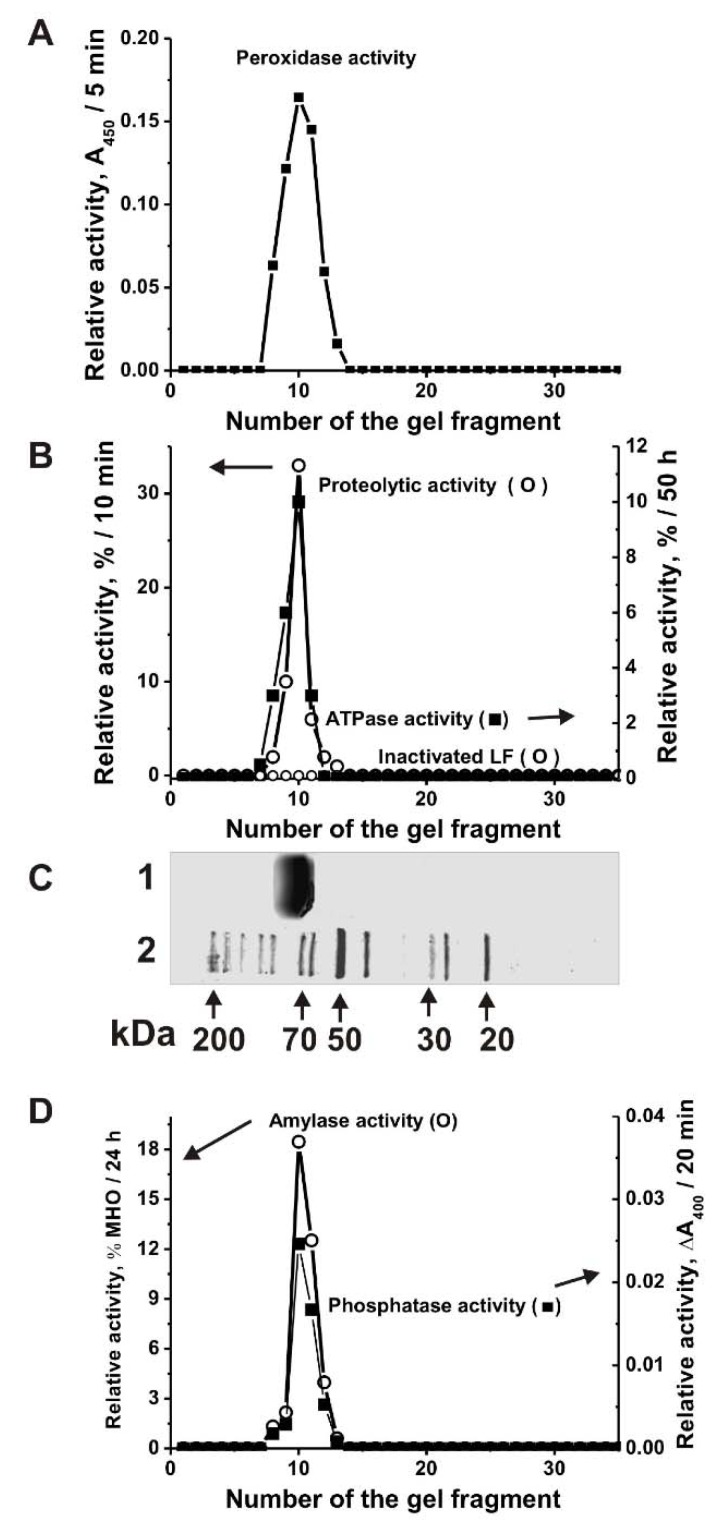
Strict criteria proving that the catalytic activities are intrinsic properties of cow LF. The relative peroxidase (**A**), proteolytic and ATPase (**B**), amylase and phosphatase (**D**) activities were revealed using the 20 µL of extracts of 2–3-mm many gel fragments of one longitudinal slice corresponding to LF_mix_ before and after its inactivation. The longitudinal control slices of the same gels were stained with collide silver (**C**): Lane 1 corresponds to LF_mix_, while Lane 2 shows the positions of molecular mass markers. The average error of determination of the initial rate from two experiments did not exceed 10–15%. For details, see Materials and Methods.

It is known that LFs of human milk and blood have several enzymatic activities [19]. It has been previously shown that similar to LF from human milk cow lactoferrin also possesses DNase and RNase activities [34]. In this article, we have shown that cow LF_mix_ also possesses five other enzymatic activities: Oxidoreductase, amylase, protease, phosphatase and ATPase. For the irrefutable assignment of all these five activities to LF and to exclude artefacts, the LF_mix_ was subjected to SDS-PAGE. All the catalytic activities were detected after extraction of proteins from many separated gel slices (Figure 2), the position of intact LF_mix_ is shown in Figure 2C, lane 1. The SDS-PAGE assay showed the absence of the activities after LF_mix_ boiling. In principle, it was impossible to exclude that even after affinity purification on anti-LF-Sepharose; LF preparations may contain impurities of any enzymes. However, before boiling SDS-PAGE peaks of all activities correspond to the protein band of only LF_mix_ (Figure 2). It should be mentioned that all classic bovine and human enzymes including proteases (24–25 kDa), metalloproteases (50–60 kDa), catalases and peroxidases (22–59 kDa), alkaline phosphatases (36.2–57.2 kDa), amylases (57–58 kDa), and ATPases (23–30 or 89–1338 kDa) possess usually lower or higher molecular masses than LF (~80 kDa) [41]. If the LF preparation contained impurities of any of these enzymes, then their peaks of the activities would be lower or higher than that for LF. Additionally, in the case of the formation of LF complexes with any of these enzymes, the peaks of catalytic activities would be located on the gel higher than that for LF. Thus, the coincidence of the peaks of all five activities only with the position on the gel of only lactoferrin showed that all five activities (protease, oxidoreductase, amylase, phosphatase, and ATPase) belong directly to lactoferrin, and not to any possible impurities of enzymes with these activities.

### 3.3. Relative Activities of Cow Lactoferrin in the Hydrolysis of Different Substrates

**Figure 3:**
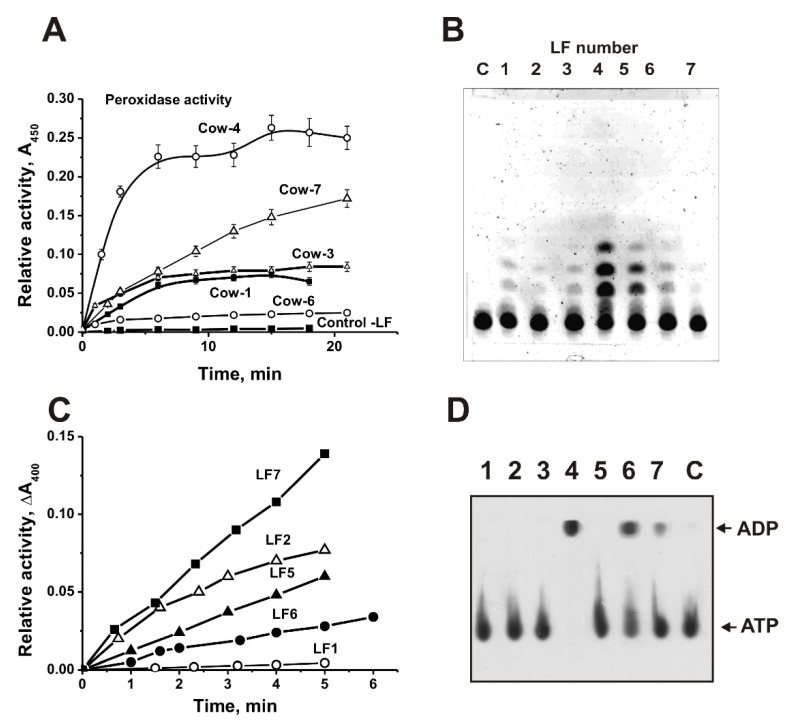
Comparison of enzymatic activities of several different preparations of cow LFs. Typical examples of the time dependence of 0.2 mg/mL diaminobenzidine (DAB) oxidation by several LF preparations (1 µM); curve control corresponds to the incubation of the substrate without LFs (**A**). Thin layer chromatography (TLC) analysis of 3 mM maltoheptaose hydrolysis for 24 h in the presence of seven different LFs (1 µM) (**B**). Lane C corresponds to the substrate incubated alone. The time dependence of the hydrolysis of *para*-nitrophenyl phosphate (5 mM; change in A_400_) by several LF preparations (**C**). Line C corresponds to *para*-nitrophenyl phosphate incubated alone. TLC analysis of ATP (0.3 mM) hydrolysis for 48 h by seven various LFs; lane C corresponds ATP incubated without LFs (**D**). For details, see Materials and Methods.

We have compared the relative activities of seven individual cows LF1–LF7 preparations in the transformation different substrates. It was shown that seven LFs are active in the oxidation of DAB in the presence of H_2_O_2_. All seven LFs demonstrated different peroxidase activity (for example, Figure 3A). The data are summarized in Table 1. The relative activities of seven different LFs significantly varied from 1.4 to 18.1 µmol DAB/1 h/1 mg LF (specific units); average value 6.1 ± 5.8 units (Table 1).

All seven LF preparations efficiently hydrolyze maltoheptaose (Figure 3B). Firstly, the efficiency of maltoheptaose hydrolysis by LFs was calculated as a percent from the substrate transition to its hydrolyzed products. Then, the activity was expressed as µmol maltoheptaose/1 h/1 mg LF. The relative amylase activities of LF varied from 25.6 to 118.4 units; the average value is 75.7 ± 29.6 µmol maltoheptaose/1 h/1 mg LF (Table 1). 

Seven LF preparations possess phosphatase activity; they efficiently hydrolyze *para*-nitrophenyl phosphate (Figure 3C). The relative phosphatase activities of LF varied from 0.011 to 0.091 units; average value is 0.038 ± 0.026 nM *p*NPP/1 h/1 mg_LF (Table 1). 

Earlier, it was shown that all LF preparations from the human milk of each of 30 mothers have ATPase activity [19]. Figure 3D shows that LF_mix_ also possesses ATPase activity. However, only three preparations of seven individual LFs demonstrated a reliably tested ATPase activity varying from 0.0016 to 0.016 µmol ATP/1 h/1 mg LF (Figure 3D; Table 1).

Protease activity of cow LF preparations was estimated using azocasein as the substrate due to the increase of reaction mixture optical density at 436 nm (∆A_436_) in the result of the elimination of azo dye from casein. The specific activity was first expressed as the increase in ∆A_436_ of azo dye after azocasein incubation with LFs. We have estimated a relative protease activity of seven LFs in the absence of external metal ions (Table 1). Depending on the preparation, the relative proteolytic activity varied in the range of 0.72–2.2 units: The average value 1.1 ± 0.51 units (A_436_/1 h/1 mg LF).

According to X-ray data, two LF lobes connected by a very flexible amino acid spacer [1]. Therefore, LF molecules are extremely conformationally flexible, and the protein functional state can be influenced not only by iron ions [1] but also by other metal ions and different ligands like DNA, RNA, polyanions, etc. [42].

**Figure 4:**
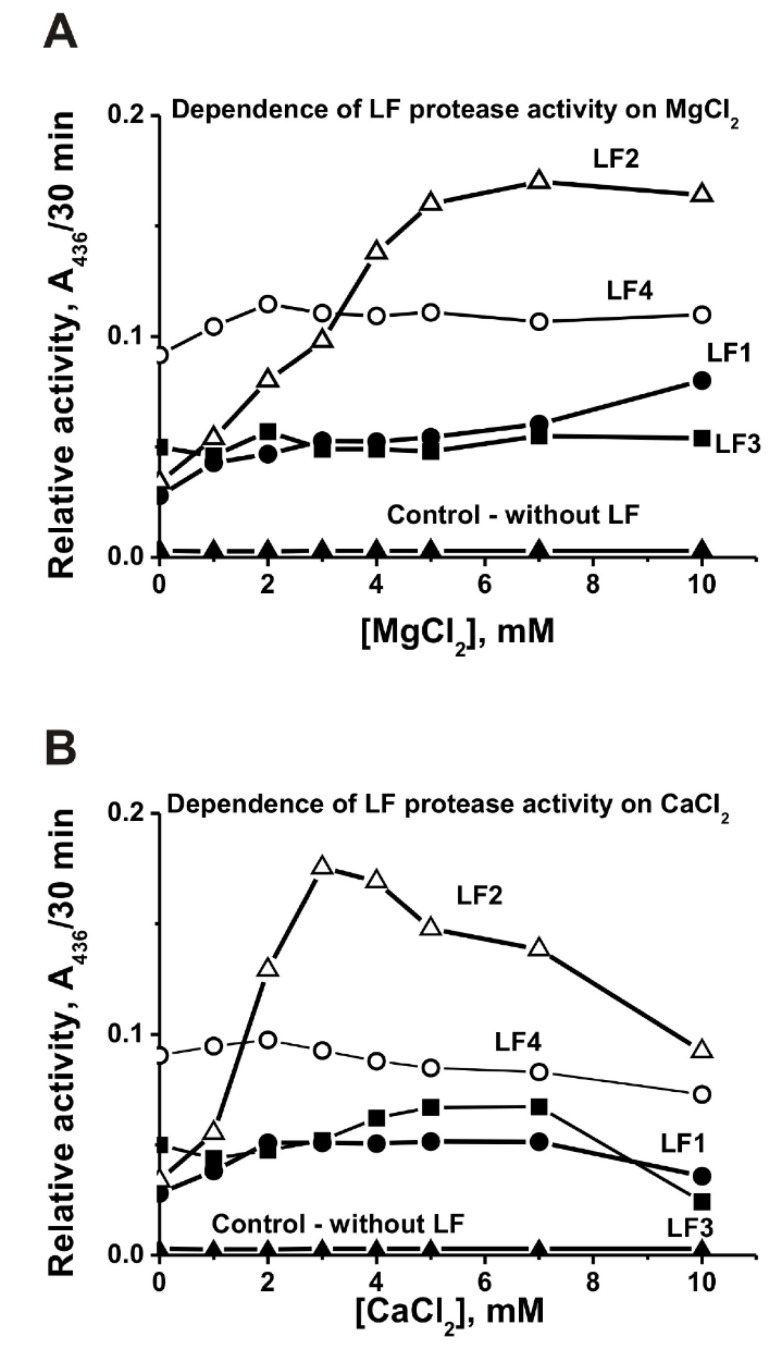
Typical examples of the dependences of proteolytic activity in the hydrolysis of azocasein for 30 min by four LF preparations (2.7 × 10^−7^ M) on the concentration of MgCl_2_ (**A**) and CaCl_2_ (**B**); control—incubation of azocasein without LFs (**A** and **B**).

Taking this into account we tried to analyze effects of Mg^2+^ and Ca^2+^ ions on the relative protease activity of LF preparations. Several typical examples dependences of the RAs on the concentrations of Mg^2+^ and Ca^2+^ are given in Figure 4A,B. One can see that the addition of Mg^2+^ ions leads to a remarkable increase of LF protease activity in the case of cows 1, 2, and 4, but does not substantially change this activity of the protein of the 3rd cow (Figure 4A). Near similar situation was observed after addition of Ca^2+^ ions (Figure 4B).

## 4. Discussion

It was shown earlier, that human LF possesses DNase, RNase, phosphatase, amylase, and ATPase activities [19], while cow lactoferrin have DNase and RNase activities [34]. For the first time here we have shown that LFs from the milk of cows of different breeds possess peroxidase, amylase, phosphatase, protease, and ATPase activities. Earlier, peroxidase and protease activities did not analyze in the case of human LFs [19]. It should be noted that only the protease (1.1 ± 0.51 A_436_/1 h/1 mg LF) and amylase (75.7 ± 29.6 µmol MHO/1 h/1 mg LF) activities of LF preparations from the milk of different cows are to some extent comparable (Table 1). All other activities of LF preparations are very different depending on the cows: Peroxidase (1.4–18.1; average 6.1 ± 5.8 µmol DAB/1 h/1 mg LF), ATPase (0–1.6 × 10^−2^; average (0.35 ± 0.60) × 10^−2^ µmol ATP/h/1 mg LF), phosphatase (0.011–0.091; average 0.038 ± 0.026 nM *p*NPP/h/1 mg_LF) (Table 1). It was shown that all human LFs possess high ATPase activity [19]. It is interesting that only three of seven cow LF preparations have authentically tested ATPase activity (Figure 3; Table 1).

It should be noted that the relative activity of LFs from cow’s milk is significantly less than that of the corresponding canonical enzymes. At the same time, in contrast to minor canonical enzymes lactoferrin is a major milk protein. 

Significant differences in the relative levels of all five catalytic activities of LF preparations from seven cows of different breeds can be associated with various factors including first of all genetic ones. 

It was shown, that catalytic activities of human LF may contribute to its protective functions due to LF-dependent hydrolysis of alien components of viruses and bacteria [38,39]. Bovine LF oral administration LF leads to host protection of patients from infections [3,14]. In principle, several catalytic activities of cow LF may contribute to its protective functions. However, in the case of LF preparations as medicinal drugs, it is necessary to search for cows whose milk contains this protein having highest of all different catalytic activities.

The nature of the formation of several active centers of human and cow LFs so far remains unclear. However, there are several potential ways to implement such lactoferrin centers. As shown by the example of human LF, some centers exist at the level of the monomeric molecules of this protein [20,35,43]. For example, it was shown that nucleic acid (DNA and RNA) and ATP binding sites are localized in two different N- and C-lobes of monomeric LF molecules respectively [43]. Other active centers can also exist initially at the level of individual LF molecules or be for example formed in the processes of their posttranslational modification or oligomerization processes. Specific conformations of monomeric LF can be induced by different ligands, which may modulate its enzymatic activities. For example, binding of DNA is not a rapid process while such ligands as ATP or NAD, significantly accelerating this process [25].

The nature of the interactions between LF molecules leading to its oligomers remains unknown. It was shown that the main oligomeric forms of LF are dimer and tetramer [44]. However, various ligands (DNA, nucleotides and oligosaccharides) of LF stimulate the formation of its oligomeric forms containing up to 10 monomers [43]. During the formation of different oligomeric forms, LF molecules can bind to each other in different ways, forming various new catalytic centers at the junction of the subunits. The biological functions and catalytic activities of LF oligomeric forms have not yet been studied, but they may be important for understanding of the protein multifunctionality. 

The LF molecule contains two potential glycosylation sites, the level of glycosylation of different protein molecules varies and they can contain hexose, mannose, hexoseamines, or other saccharides and may also differ in the level of phosphorylation [45]. Different types of LF glycosylation can also, in principle, alter the biological and enzymatic functions of individual protein molecules. It cannot be excluded that some sugar residues may take part in the formation of the active centers of some individual LF molecules. In this regard, the following data should be noted. During chromatography on Blue Sepharose, the homogeneous LF preparation was divided into four fractions [20]. The first fraction with the lowest affinity for Blue Sepharose did not possess catalytic activities analyzed, the second and third fractions effectively hydrolyzed DNA and RNA, while the fourth fraction having the highest affinity for the sorbent hydrolyzed only ATP. These data may indicate that LF molecules modified by various ways may have different catalytic functions.

The relative level of cow LF catalytic activities found by us in vitro can in principle be substantially lower than directly in the milk. Thus, the protease activity of some LF preparations increases in the presence of Mg^2+^ and Ca^2+^ ions (Figure 4). Considering the relatively small size of the monomer and its polyfunctionality one can suggest that different functions and catalytic activities of cow and human LFs might be probably realized due to their different modified molecules and various oligomeric forms, which interconversion could be controlled by ATP, metal ions and/or other various ligands. 

## 5. Conclusions

Thus, it was shown for the first time that the lactoferrin of fresh cow milk has several enzymatic functions. The discovery of five enzymatic activities may contribute in the future to understanding the nature of LF polyfunctional functions in fresh milk.

## Figures and Tables

**Table 1:** Relative activities of seven cow LFs in the catalysis of five different chemical reactions *.

Cow and LF number	Peroxidase Activity, µmol DAB/1 h/1 mg LF	Azocasein Hydrolysis Activity, A_436_/1 h/1 mg	Amylase Activity, µmol MHO/1 h/1 mg LF	Phosphatase, nM *p*NPP/1 h /1 mg_LF	ATPase Activity, (µmol ATP/1 h/1 mg LF) × 10^2^
1	3.1 ± 0.2	0.72 ± 0.04	62.4 ± 4.0	0.011	~0.0
2	3.6 ± 0.2	0.87 ± 0.05	65.6 ± 5.0	0.04	~0.0
3	9.3 ± 0.4	1.2 ± 0.01	80.0 ± 6.0	0.03	~0.0
4	18.1 ± 0.8	2.2 ± 0.01	118.4 ± 7.0	0.042	1.6 ± 0.1
5	2.5 ± 0.2	1.0 ± 0.01	100.8 ± 6.0	0.036	~0.0
6	1.4 ± 0.1	0.97 ± 0.06	76.8 ± 3.0	0.016	0.72 ± 0.05
7	4.7 ± 0.3	0.72 ± 0.05	25.6 ± 1.5	0.091	0.16 ± 0.01
Average value	6.1 ± 5.8	1.1 ± 0.51	75.7 ± 29.6	0.038 ± 0.026	0.35 ± 0.60

* The average values of three independent experiments are given as average mean ± average deviation.
